# Association of conventional haemostasis and coagulation tests with the risk of acute upper gastrointestinal bleeding in liver cirrhosis: a retrospective study

**DOI:** 10.1093/gastro/gov059

**Published:** 2015-12-15

**Authors:** Jing Li, Xingshun Qi, Han Deng, Ying Peng, Lichun Shao, Jiaxin Ma, Xiaolin Sun, Hongyu Li, Xiaozhong Guo

**Affiliations:** ^1^Liver Cirrhosis Study Group, Department of Gastroenterology, General Hospital of Shenyang Military Area, Shenyang, China; ^2^Postgraduate College, Dalian Medical University, Dalian, China; ^3^Department of Gastroenterology, No. 463 Hospital of Chinese PLA, Shenyang, China

**Keywords:** coagulation, bleeding, liver cirrhosis, platelets, prothrombin

## Abstract

**Objective:** A retrospective study was performed to compare the difference in platelet count (PLT), prothrombin time (PT), international normalized ratio (INR), and activated partial thromboplastin time (APTT), between cirrhotic patients with and without acute upper gastrointestinal bleeding (AUGIB) or acute oesophageal variceal bleeding (AEVB).

**Methods:** Between January 2012 and June 2014, a total of 1734 cirrhotic patients were enrolled and were classified into ‘AUGIB’ (*n = *497) and ‘no AUGIB’ (*n = *1237) groups according to their disease history. They were further divided into ‘AEVB’ (*n = *297) and ‘no AEVB’ (*n = *1259) groups according to the endoscopic findings. Additionally, 178 patients with AUGIB were not assigned to either the ‘AEVB’ or ‘no AEVB’ groups due to the absence of any endoscopic findings.

**Results:** Compared with the ‘no AUGIB’ group, the ‘AUGIB’ group had similar PLT (99.99 ± 89.90 *vs.*101.47 ± 83.03; *P = *0.734) and APTT (42.96 ± 15.20 *vs.*43.77 ± 11.01; *P = *0.219), but significantly higher PT (17.30 ± 5.62 *vs.*16.03 ± 4.68; *P < *0.001) and INR (1.45 ± 0.69 *vs.*1.31 ± 0.59; *P < *0.001). A lower PT was independently associated with the absence of AUGIB (OR = 0.968; 95% CI: 0.942–0.994). Compared with the ‘no AEVB’ group, the ‘AEVB’ group had significantly lower PLT (86.87 ± 62.14 *vs.*101.74 ± 83.62; *P = *0.004) and APTT (40.98 ± 7.9 *vs.*43.72 ± 10.97; *P < *0.001), but similar PT (16.53 ± 3.71 *vs.*16.04 ± 4.68; *P = *0.088) and INR (1.35 ± 0.41 *vs.*1.31 ± 0.59; *P = *0.225). A higher PLT was independently associated with the absence of AEVB (OR = 1.004; 95% CI: 1.002–1.006; *P = *0.001).

**Conclusions:** PLT was associated with the occurrence of portal hypertension-related bleeding in liver cirrhosis.

## Introduction

Traditionally, a patient with liver cirrhosis is often at a high risk of bleeding, especially portal hypertension-related variceal bleeding [1, 2]. Recently, our systematic review has confirmed that about 1% of cirrhotic patients have a diagnosis of venous thromboembolism (VTE) on admission [3]. It appears that cirrhotic patients have a higher prevalence of VTE than healthy controls [4]. Additionally, portal vein thrombosis is relatively frequent in cirrhotic patients, especially in those with more severe liver dysfunction [5]. Except for the evidence from epidemiological studies, experimental studies have also found that such patients had a pro-coagulant tendency, including lower protein C and higher factor VIII etc. [6, 7]. Re-balance between coagulation and anticoagulation has been proposed in liver cirrhosis, but it is easily upset, so that bleeding and thrombosis events develop [8]. Regular surveillance of haemostatic factors and coagulation status is necessary to evaluate the possibility of bleeding and thrombosis. This knowledge will alert the physicians and patients to the relevant risks.

The most common diagnostic tests for haemostasis and coagulation in cirrhotic patients include platelet count (PLT), prothrombin time (PT), international normalized ratio (INR), and activated partial thromboplastin time (APTT). They are inexpensive, rapid, and widely used to evaluate the bleeding tendency in our clinical practices. Briefly, if PT, INR, and APTT were elevated or PLT were decreased, the risk of bleeding would be increased. However, some recent perspectives suggested that PT and INR could reflect the severity of liver dysfunction and risk of mortality in acute or chronic liver diseases, but not the risk of bleeding [9]. PLT was significantly associated with hepatic venous pressure gradient in cirrhotic patients who had never bled [10]. Reduced PLT could reflect the presence of portal hypertension, especially its related hypersplenism. In this study, we have explored the association of the four readily available haemostasis and coagulation tests with the occurrence of acute upper gastrointestinal bleeding (AUGIB) and acute oesophageal variceal bleeding (AEVB) in liver cirrhosis.

## Patients and methods

### Patients

All cirrhotic patients who were consecutively admitted to our hospital between January 2012 and June 2014 were potentially eligible for this retrospective study. In our hospital, a diagnosis of liver cirrhosis was often established by the clinical presentations (decompensated events), liver function tests (total bilirubin, albumin, etc.), and abdominal ultrasound and CT scans (liver contour, spleen size, portal vein, and oesophageal varices) [11, 12]. Liver biopsy was considered if a diagnosis of liver cirrhosis was ambiguous. Exclusion criteria were (i) that patients were diagnosed with malignancy and (ii) that regular coagulation test results, such as PLT, PT, INR, and APTT, were absent. This study was approved by the Ethics Committee of our hospital [number k(2015)15]. The informed consent was waived.

### Data collection

Information from all patients with a diagnosis of liver cirrhosis was searched by our hospital Department of Information. All clinical records were carefully reviewed to identify the patients with a diagnosis of malignancy, especially HCC. All data were collected from the electronic medical charts. The primary data items included sex, age, aetiology of liver diseases, ascites, hepatic encephalopathy, varices, laboratory tests, Child-Pugh class/score [13], and model for end-stage liver disease (MELD) score [14].

### Definitions and classifications

AUGIB was defined as a new onset of haematemesis or melaena within 5 days prior to admission at our hospital. AEVB was defined as an episode of AUGIB with evidence of oesophageal varices on upper gastrointestinal endoscopy if other possible sources, such as ulcer, had been excluded. Thus, the patients were divided into two groups: (i) patients with AUGIB; and (ii) patients without AUGIB. Additionally, they were further classified into two subgroups: (a) patients with AEVB and (b) patients without AEVB. All episodes of AUGIB and AEVB were evaluated by two investigators (YP and JL), their accuracy was then confirmed by another two investigators (HD and XQ). Any disagreement among them was resolved by discussion. The grades of ascites and hepatic encephalopathy were evaluated according to the current guidelines and consensus.

### Statistical analysis

Continuous variables were expressed as mean ± standard deviation or median (range), and were compared by using the independent sample *t*-test or non-parametric Wilcoxon test. Categorical variables were expressed as frequency (percentage), and were compared by using the Chi-squared test. Box plots were also drawn to show the statistical differences between groups in the PLT, PT, INR, and APTT. All variables that were statistically significantly associated with AUGIB/AEVB in univariate analyses were entered into multivariate analyses. Independent variables were reported with odds ratios (ORs) with 95% confidence intervals (CIs). A two-tailed *P-*value of <0.05 was considered statistically significant. All statistical analyses were employed by using SPSS Statistics 17.0.

## Results

### Overall analysis (with and without AUGIB)

During the enrolment period, a total of 1734 patients with a diagnosis of liver cirrhosis were eligible for our study. The patients’ characteristics are shown in Table 1. Among them, 497 and 1237 patients presented with and without AUGIB, respectively. A majority of them were male (66.6%), had viral hepatitis with or without alcohol abuse (46.3%), and were in Child-Pugh classes A and B (79.5%).

**Table 1.** Comparison between patients with and without acute upper gastrointestinal bleeding (AUGIB)

Variables	Total	With AUGIB	Without AUGIB	*P-* value
	(*n = *1734)	(*n = *497)	(*n = *1237)	
Gender (male/female)	1154/580	333/164	821/416	0.801
Age (years)	56.23 ± 12.06	55.69 ± 12.02	56.45 ± 12.08	0.232
Aetiology of liver diseases				0.336
Viral hepatitis	595 (34.3)	180 (36.2)	415 (33.5)	
Alcohol	456 (26.3)	124 (24.9)	332 (26.8)	
Viral hepatitis + Alcohol	144 (8.3)	35 (7.0)	109 (8.8)	
Others	208 (12.0)	68 (13.7)	140 (11.3)	
Unknown	331 (19.1)	90 (18.1)	241 (19.5)	
Ascites	854 (49.3)	232 (46.7)	622 (50.3)	0.175
Hepatic encephalopathy	115 (6.6)	31 (6.2)	84 (6.8)	0.676
Varices				**<0.001**
No	99 (5.7)	22 (4.4)	77 (6.2)	
Mild	30 (1.7)	6 (1.2)	24 (1.9)	
Moderate	84 (4.8)	37 (7.4)	47 (3.8)	
Severe	475 (27.4)	254 (51.1)	221 (17.9)	
Not evaluated by endoscopy	1046 (60.3)	178 (35.8)	868 (70.2)	
Laboratory tests				
Haemoglobin (g/L)	94.86 ± 30.07	73.48 ± 21.94	103.45 ± 28.59	**<0.001**
Platelet (×10^9^/L)	101.05 ± 85.03	99.99 ± 89.90	101.47 ± 83.03	0.734
Total bilirubin (μmol/L)	20.85 (1.9–809.8)	19.7 (3.3–250.8)	21.95 (1.9–809.8)	**<0.001**
Albumin (g/L)	32.10 ± 6.87	30.18 ± 6.66	32.88 ± 6.80	**<0.001**
Creatinine (µmol/L)	59 (2.82–1473)	58.8 (20–919)	59 (2.82–1473)	**0.001**
Prothrombin time (s)	16.39 ± 5.00	17.30 ± 5.62	16.03 ± 4.68	**<0.001**
APTT (s)	43.53 ± 12.36	42.96 ± 15.20	43.77 ± 11.01	0.219
INR	1.35 ± 0.62	1.45 ± 0.69	1.31 ± 0.59	**<0.001**
Child-Pugh class				0.115
A	627 (36.2)	168 (33.8)	459 (37.1)	
B	751 (43.3)	234 (47.1)	517 (41.8)	
C	288 (16.6)	75 (15.1)	213 (17.2)	
Not evaluated	68 (3.9)	20 (4.0)	48 (3.9)	
Child-Pugh score	7 (5–15)	7 (5–14)	7 (5–15)	0.615
MELD score	5.8 (-14.3–51.6)	5.4 (-7.5–39.4)	5.8 (-14.3–51.6)	0.115

Values are presented as mean ± SD, median (range) or *n* (%). APTT = activated partial thromboplastin time; INR = ; MELD = model for end stage liver disease

Patients with AUGIB had significantly lower haemoglobin (Hb) than those without. PLT was not significantly different between patients with and without AUGIB (99.99 ± 89.90 *vs.*101.47 ± 83.03; *P* *=* 0.734).

Patients with AUGIB had significantly lower total bilirubin (TBIL), albumin (ALB), and creatinine (Cr) levels than those without AUGIB. PT and INR were significantly higher in patients with AUGIB than in those without (17.30 ± 5.62 *vs.*16.03 ± 4.68; *P* *<* 0.001; 1.45 ± 0.69 *vs.*1.31 ± 0.59; *P* *<* 0.001) and APTT was not significantly different between the two groups (42.96 ± 15.20 *vs.*43.77 ± 11.01; *P* *=* 0.219). Child-Pugh and MELD scores were not significantly different between patients with and without AUGIB.

Haemoglobin, TBIL, ALB, Cr, and PT were included in the multivariate analysis (Table 2). A lower PT was independently associated with the absence of AUGIB (OR = 0.968, 95% CI: 0.942–0.994; *P* *=* 0.015).

**Table 2.** Multivariate analysis of factors associated with acute upper gastrointestinal bleeding

Variables	*P-*value	Odds ratios	95% confidential interval	
			Lower limit	Upper limit
Haemoglobin	<0.001	1.044	1.038	1.050
Total bilirubin	<0.001	1.007	1.004	1.011
Albumin	0.062	1.020	0.999	1.042
Creatinine	<0.001	1.005	1.003	1.008
Prothrombin time	0.015	0.968	0.942	0.994

## Subgroup analysis (with and without AEVB)

In 178 patients with AUGIB, upper gastrointestinal endoscopy was not performed to evaluate the presence of varices. Among the remaining 319 AUGIB patients undergoing upper gastrointestinal endoscopy, 22, 6, 37, and 254 patients had no, mild, moderate, and severe varices, respectively. The characteristics of patients with and without AEVB are shown in Table 3.

**Table 3.** Comparison between patients with and without acute variceal bleeding (AEVB)

Variables	With AEVB	Without AEVB	*P-*value
	(*n = *297)	(*n = *1259)	
Gender (male/female)	199/98	829/430	0.705
Age (years)	54.37 ± 11.56	56.42 ± 12.12	**0.008**
Aetiology of liver diseases			0.529
Viral hepatitis	108 (36.4)	428 (34.0)	
Alcohol	70 (26.2)	336 (26.7)	
Viral hepatitis + alcohol	28 (9.4)	109 (8.7)	
Others	40 (13.5)	141 (11.2)	
Unknown	51 (17.2)	245 (19.5)	
Ascites	135 (45.5)	626 (49.7)	0.186
Hepatic encephalopathy	10 (3.4)	85 (6.8)	**0.028**
Varices			**<0.001**
No	0 (0.0)	99 (7.9)	
Mild	6 (2.0)	24 (1.9)	
Moderate	37 (12.4)	47 (3.7)	
Severe	254 (85.5)	221 (17.6)	
Not evaluated	0 (0.0)	868 (68.9	
Laboratory tests			
Haemoglobin (g/L)	74.13 ± 20.78	102.85 ± 28.88	**<0.001**
Platelet (×10^9^/L)	86.87 ± 62.14	101.74 ± 83.62	**0.004**
Total bilirubin (µmol/L)	18.0 (3.3–107)	21.9 (1.9–809.8)	**<0.001**
Albumin (g/L)	31.24 ± 6.35	32.83 ± 6.78	**<0.001**
Creatinine (µmol/L)	59 (28–327)	59 (2.82–1473)	**0.001**
Prothrombin time (s)	16.53 ± 3.71	16.04 ± 4.68	0.088
APTT (s)	40.98 ± 7.98	43.72 ± 10.97	**<0.001**
INR	1.35 ± 0.41	1.31 ± 0.59	0.225
Child-Pugh class			0.057
A	119 (40.1)	473 (37.6)	
B	133 (44.8)	523 (41.5)	
C	34 (11.4)	215 (17.1)	
Not evaluated	11 (3.7)	48 (3.8)	
Child-Pugh score	7 (5–13)	7 (5–15)	0.057
MELD score	5.1 (-7.4–37.6)	5.8 (-14.3–51.6)	**0.002**

Values are presented as mean ± SD, median (range) or *n* (%). APTT = activated partial thromboplastin time; INR = international normalized ratio; MELD = model for end stage liver disease

Patients with AEVB had significantly lower Hb than those without AEVB. PLT was significantly lower in patients with AEVB than in those without AEVB (86.87 ± 62.14 *vs.*101.74 ± 83.62; *P* *=* 0.004).

Patients with AEVB had significantly lower TBIL, ALB, and Cr levels than those without. PT and INR were not significantly different between the two groups (16.53 ± 3.71 *vs.*16.04 ± 4.68; *P* *=* 0.088; 1.35 ± 0.41 *vs.*1.31 ± 0.59; *P* *=* 0.225); and APTT was significantly lower in patients with AEVB than in those without it (40.98 ± 7.98 *vs.*43.72 ± 10.97; *P* *<* 0.001). Child-Pugh and MELD scores were higher in patient with AEVB than those without. The difference was statistically significant for MELD score, but not for Child-Pugh score.

Haemoglobin, PLT, TBIL, ALB, Cr, and APTT were included in the multivariate analysis (Table 4). A higher PLT was independently associated with the absence of AEVB (OR = 1.004; 95% CI: 1.002–1.006; *P* *=* 0.001).

**Table 4.** Multivariate analysis of variables associated with acute variceal bleeding

Variables	*P-*value	Odds ratios	95% confidential interval	
			Lower limit	Upper limit
Haemoglobin	<0.001	1.044	1.037	1.051
Platelet	0.001	1.004	1.002	1.006
Total bilirubin	0.011	1.008	1.002	1.014
Albumin	0.051	1.026	1.000	1.052
Creatinine	<0.001	1.008	1.004	1.011
Activated partial thromboplastin time	<0.001	1.074	1.048	1.102

## Discussion

PT/INR is a major component of Child-Pugh and MELD score [13, 14], which reflects the severity of liver dysfunction in liver cirrhosis. However, the association between PT/INR and risk of bleeding and haemostasis in liver cirrhosis has been frequently questioned. More recently, the Baveno VI Consensus Workshop on the management of portal hypertension has clearly suggested that PT/INR is not a reliable indicator of coagulation status in patients with cirrhosis [15]. This recommendation is strong and of high grade. In the present study, we attempted to retrospectively compare the difference of PT/INR and PLT between patients with and without recent bleeding. Our study found that PT/INR could reflect the occurrence of all-cause AUGIB in liver cirrhosis, but not AEVB. By contrast, PLT could reflect the occurrence of AEVB in liver cirrhosis, but not AUGIB.

AUGIB could be attributed to various aetiologies in liver cirrhosis, such as portal hypertension, peptic ulcer, and others. Regardless of any aetiologies of AUGIB, PT/INR was associated with the bleeding tendency; certainly, we had to acknowledge that the absolute difference of mean PT/INR values between the two groups was close to 1. Thus, it should be noted that PT/INR might play only a minor role in the occurrence of AUGIB.

On the other hand, AEVB was related to portal hypertension on its own. Splenomegaly and hypersplenism were also common complications of portal hypertension. Many studies have found a close association between PLT and the severity of portal hypertension [16, 17]; thus, it is more reasonable to speculate that PLT predicts the occurrence of portal hypertension-related bleeding; indeed, there was also a relatively large absolute difference of mean PLT values between the two groups.

Our study had two major strengths: first, there was a relatively large sample size; our conclusions might thus be more stable. Second, four authors (JL, YP, HD, and XQ) participated in identifying and confirming the acute bleeding episodes; thus we could more accurately evaluate the factors associated with acute bleeding episodes.

A major limitation of our study should be clearly emphasized: that its retrospective nature precluded accurate identification of the causes of all AUGIB episodes. Not all patients with AUGIB underwent endoscopic examinations. Endoscopic treatment was based on the presence of active bleeding and patients’ preferences. Patient selection bias has never been neglected; relevant data were not available in some patients. Second, we initially planned to validate our conclusions by using the data from another hospital (i.e. No. 463 Hospital of the Chinese PLA). Although two authors (LS and JM) had finished the data collection, very few patients with AUGIB at that hospital had undergone endoscopic examination. Thus, further external validation is warranted. Third, due to the retrospective nature of the study, the use of concurrent medications that may influence the PT and INR—such as warfarin or heparin—was not clearly recorded. Fourth, hepatic venous pressure gradient (HVPG) is the reference standard of portal hypertension; however, HVPG measurement is not carried out in our hospital. Thus, future studies should explore the correlation between PLT, PT/INR, and APTT with HVPG and upper gastrointestinal endoscopy examinations. Fifth, the definitions of variceal bleeding were not excellent.

In conclusion, PLT—but not PT/INR—reflected the occurrence of portal hypertension-related bleeding in liver cirrhosis. Accordingly, we would like to further ask whether oral warfarin prolongs the PT but does not increase the risk of variceal bleeding in liver cirrhosis. This question is clinically important, because anti-coagulation therapy may recanalize the thrombosed portal vein, prevent the development of portal vein thrombosis, and even improve liver function [18, 19].

### Author contributions

Jing Li reviewed the literature, wrote the protocol, collected the data, and performed the statistical analysis. Xingshun Qi designed the study, wrote the protocol, performed the statistical analysis, interpreted the data, and drafted the manuscript. Han Deng, Ying Peng, Jiaxin Ma, Lichun Shao, and Xiaolin Sun collected the data. Xiaozhong Guo and Hongyu Li gave critical comments and revised the manuscript. All authors have made an intellectual contribution to the manuscript and approved its submission.

## Funding

This study was partially supported by the grant from the National Natural Science Foundation of China (no. 81500474) and Natural Science Foundation of Liaoning Province (no. 2015020409).

*Conflict of interest statement*: none declared.

## References

[1] Garcia-Tsao G Bosch J Management of varices and variceal haemorrhage in cirrhosis. N Engl J Med 2010;362:823–32. 20200386 10.1056/NEJMra0901512

[2] Harrison PM Management of patients with decompensated cirrhosis. Clin Med 2015;15:201–3. 10.7861/clinmedicine.15-2-201 PMC4953743 25824076

[3] Qi X Ren W Guo X Epidemiology of venous thromboembolism in patients with liver diseases: a systematic review and meta-analysis. Intern Emerg Med 2015;10:205–17. 25472621 10.1007/s11739-014-1163-7

[4] Sogaard KK Horvath-Puho E Gronbaek H Risk of venous thromboembolism in patients with liver disease: a nationwide population-based case-control study. Am J Gastroenterol 2009;104:96–101. 19098856 10.1038/ajg.2008.34

[5] Qi X Han G Fan D Management of portal vein thrombosis in liver cirrhosis. Nat Rev Gastroenterol Hepatol 2014;11:435–46. 24686266 10.1038/nrgastro.2014.36

[6] Tripodi A Mannucci PM The coagulopathy of chronic liver disease. N Engl J Med 2011;365:147–56. 21751907 10.1056/NEJMra1011170

[7] Tripodi A Primignani M Chantarangkul V An imbalance of pro- *vs.*anti-coagulation factors in plasma from patients with cirrhosis. Gastroenterology 2009;137:2105–11. 19706293 10.1053/j.gastro.2009.08.045

[8] Lisman T Porte RJ Rebalanced haemostasis in patients with liver disease: evidence and clinical consequences. Blood 2010;116:878–85. 20400681 10.1182/blood-2010-02-261891

[9] Northup PG Caldwell SH Coagulation in liver disease: a guide for the clinician. Clin Gastroenterol Hepatol 2013;11:1064–74. 23506859 10.1016/j.cgh.2013.02.026

[10] Qamar AA Grace ND Groszmann RJ Platelet count is not a predictor of the presence or development of gastroesophageal varices in cirrhosis. Hepatology 2008;47: 153–59. 18161700 10.1002/hep.21941

[11] Peng Y Qi X Dai J Child-Pugh versus MELD score for predicting the in-hospital mortality of acute upper gastrointestinal bleeding in liver cirrhosis. Int J Clin Exp Med 2015;8:751–7. 25785053 PMC4358508

[12] Qi X Peng Y Li H Diabetes is associated with an increased risk of in-hospital mortality in liver cirrhosis with acute upper gastrointestinal bleeding. Eur J Gastroenterol Hepatol 2015;27:476–7. 10.1097/MEG.0000000000000324 25874528

[13] Child CG Turcotte JG Surgery and portal hypertension. Major Probl Clin Surg 1964;1:1–85. 4950264

[14] Kamath PS Kim WR The model for end-stage liver disease (MELD). Hepatology 2007;45:797–805. 17326206 10.1002/hep.21563

[15] de Franchis R Expanding consensus in portal hypertension: Report of the Baveno VI Consensus Workshop: Stratifying risk and individualizing care for portal hypertension. J Hepatol 2015;63:743–52. 26047908 10.1016/j.jhep.2015.05.022

[16] Zaman A Hapke R Flora K Factors predicting the presence of oesophageal or gastric varices in patients with advanced liver disease. Am J Gastroenterol 1999;94:3292–6. 10566732 10.1111/j.1572-0241.1999.01540.x

[17] Madhotra R Mulcahy HE Willner I Prediction of oesophageal varices in patients with cirrhosis. J Clin Gastroenterol 2002;34:81–5. 11743252 10.1097/00004836-200201000-00016

[18] Qi X De Stefano V Li H Anticoagulation for the treatment of portal vein thrombosis in liver cirrhosis: A systematic review and meta-analysis of observational studies. Eur J Intern Med 2015;26:23–9. 25566699 10.1016/j.ejim.2014.12.002

[19] Villa E Camma C Marietta M Enoxaparin prevents portal vein thrombosis and liver decompensation in patients with advanced cirrhosis. Gastroenterology 2012;143:1253–60.e1–4. 22819864 10.1053/j.gastro.2012.07.018

